# Genetic insights into the age-specific biological mechanisms governing human ovarian aging

**DOI:** 10.1016/j.ajhg.2023.07.006

**Published:** 2023-08-04

**Authors:** Sven E. Ojavee, Liza Darrous, Marion Patxot, Kristi Läll, Krista Fischer, Reedik Mägi, Zoltan Kutalik, Matthew R. Robinson

**Affiliations:** 1 Department of Computational Biology, University of Lausanne, Lausanne, Switzerland; 2 Swiss Institute of Bioinformatics, Lausanne, Switzerland; 3 University Center for Primary Care and Public Health, Lausanne, Switzerland; 4 Estonian Genome Centre, Institute of Genomics, University of Tartu, Tartu, Estonia; 5 Institute of Mathematics and Statistics, University of Tartu, Tartu, Estonia; 6 Institute of Science and Technology Austria, Klosterneuburg, Austria

**Keywords:** genome-wide association study, age at natural menopause, age-specific genetic effect, aging, age at onset, time to event, common complex disease, disease etiology, significance testing, interaction

## Abstract

There is currently little evidence that the genetic basis of human phenotype varies significantly across the lifespan. However, time-to-event phenotypes are understudied and can be thought of as reflecting an underlying hazard, which is unlikely to be constant through life when values take a broad range. Here, we find that 74% of 245 genome-wide significant genetic associations with age at natural menopause (ANM) in the UK Biobank show a form of age-specific effect. Nineteen of these replicated discoveries are identified only by our modeling framework, which determines the time dependency of DNA-variant age-at-onset associations without a significant multiple-testing burden. Across the range of early to late menopause, we find evidence for significantly different underlying biological pathways, changes in the signs of genetic correlations of ANM to health indicators and outcomes, and differences in inferred causal relationships. We find that DNA damage response processes only act to shape ovarian reserve and depletion for women of early ANM. Genetically mediated delays in ANM were associated with increased relative risk of breast cancer and leiomyoma at all ages and with high cholesterol and heart failure for late-ANM women. These findings suggest that a better understanding of the age dependency of genetic risk factor relationships among health indicators and outcomes is achievable through appropriate statistical modeling of large-scale biobank data.

## Teaser

Many phenotypic characteristics and the risk of common complex disease change across life, reflecting a range of different age-specific biological processes. Here, we show that 74% of 245 genome-wide significant genetic associations with age at natural menopause show a form of age-specific effect.

## Introduction

Age at onset and time-to-event observations are among the most important traits of interest in cohort studies of age-related diseases as they are critical to gain insight into the genetics of disease development and progression.^1^^,^^2^ The underlying etiology of age-related outcomes likely reflects a variety of biological processes that are triggered at different stages of life, long before the onset of observable symptoms. As a result, the underlying genetic propensity for outcomes may vary with age and depend upon different sets of genetic risk factors at different time points, reflecting the range of underlying molecular mechanisms that shape the onset distribution. Therefore, identifying the genetic variants associated with onset at different stages of life will improve our understanding of disease progression.

**Figure 1:**
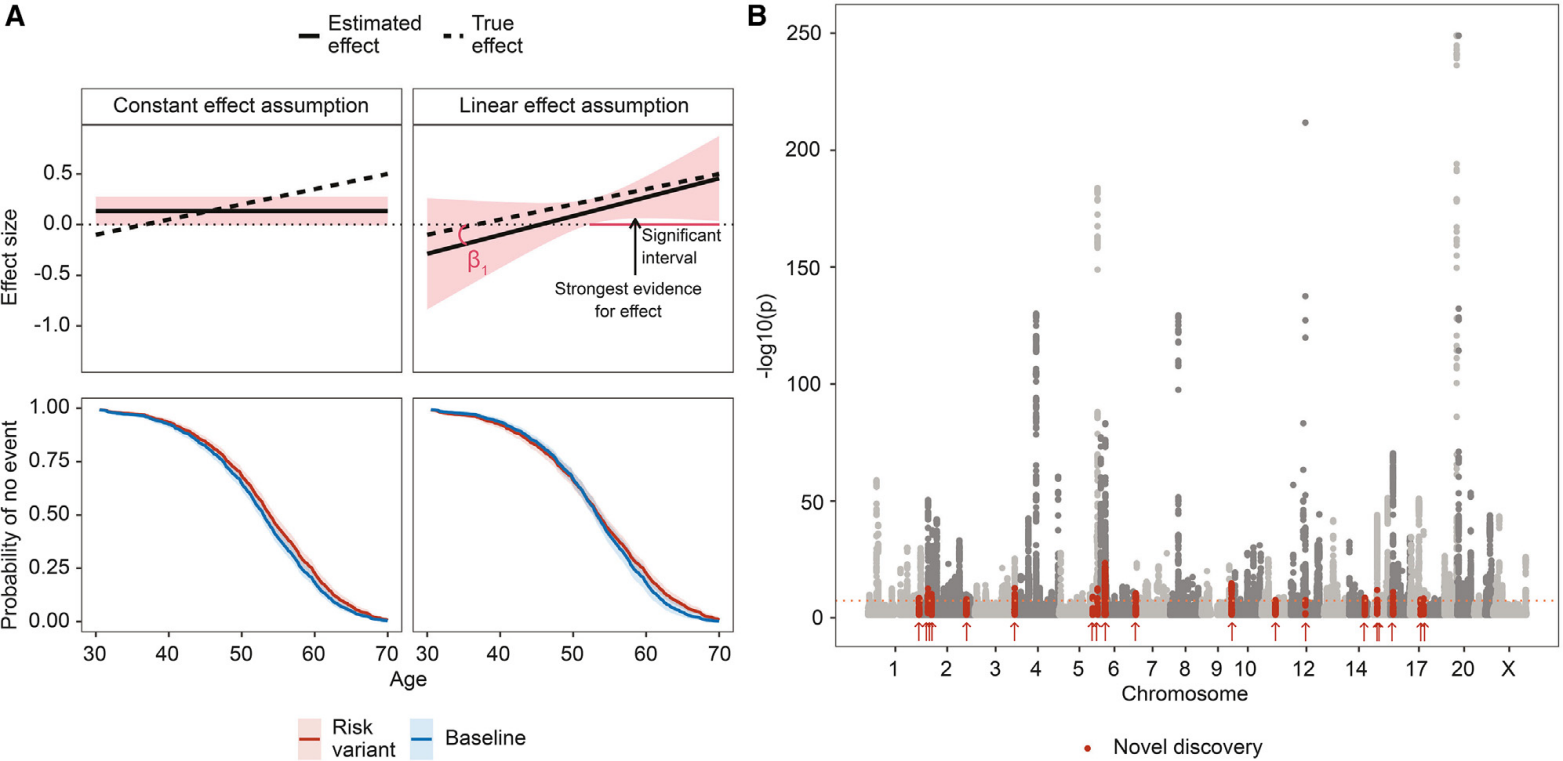
Statistical model description and previously unreported discoveries (A) The CAMP model enables a more flexible and accurate description of the SNP effect size by introducing a slope term. The linear change model enables three example questions to be addressed: (1) what is the interval at which there is a significant effect on the trait (“significant interval”)? (2) At which age is there strongest evidence for an effect (“strongest evidence for effect”)? (3) Is the slope ($\beta_{1}$) significantly different from zero (“$\beta_{1}$”)? Even though a more complex model can result in generally wider confidence intervals, it can still result in a more accurate representation of the effect size, often accompanied by higher statistical power. By estimating the effect-size change, it is also possible to accurately determine trends, which constant effect assumptions cannot capture. The lower two panels represent corresponding survival function estimates showing the probability that an individual has not experienced an event. We observe that under linear effect assumption, the survival functions can cross, which is not possible with the constant effect assumption. (B) 19 previously unreported discoveries for ANM from the CAMP model across the UK and Estonian Biobank data. The coral line indicates the genome-wide significance level of $5\times 10^{-8}$. Arrows indicate the positions of the previously unreported discoveries.

Here, we seek to test the hypothesis that genetic propensity for age at onset is age specific by focusing on the most commonly experienced timing-related phenotype in the human population, age at natural menopause (ANM). Menopause is the permanent cessation of the menstrual cycle in women following the loss of ovarian function and occurs at an average age of 51 years, with 4% of the female population experiencing early menopause prior to age 45. Current evidence suggests that early menopause is associated with a risk for cardiovascular disease^3^ and osteoporosis,^4^ and late menopause is associated with a risk for breast cancer.^5^ Recent genomic studies find ∼50% of menopausal timing variation is attributable to genetic markers^6^ that are linked to regulation of DNA repair and immune function.^7^^,^^8^^,^^9^ However, previous analyses make strong assumptions that genetic effects are constant throughout life (Figure 1A). By modeling the quantitative genetic basis of ANM in a way that enables detection of the age at which genetic risk factors have the greatest influence, we report evidence for widespread age-specific genetic effects underlying population-level variation in ovarian aging in both the UK and Estonian Biobank data.

## Material and methods

### Marginal age-specific mixed Cox proportional hazards model

We present a marginal Cox age-specific mixed proportional hazards (CAMP) model paired with a unique significance-testing framework. Following the success of many genome-wide association studies (GWASs), more attention has been attributed to better characterizing SNP effects under different environmental conditions, leading to genotype-covariate analyses.^10^ For a continuous trait, one of the simplest ways to model this type of interaction would be to include a linear interaction term. For example, to estimate the impact of age on SNP effects, we could write the model as

(Equation 1) $$y_{i}=\mu +x_{ij}\beta_{j}^{0}+x_{ij}\left(t_{i}-t_{0}\right)\beta_{j}^{1}+\varepsilon_{i}\text{,}$$

where (for individual *i* and SNP *j*) $y_{i}$ is a continuous trait, *μ* is the intercept, $x_{ij}$ is the SNP value, $\beta_{j}^{0}$ is the SNP effect at time $t_{0}$, $t_{i}$ is the age when $y_{ij}$ is measured, $\beta_{j}^{1}$ is the linear effect of age, and $\varepsilon_{i}$ is the residual variance. However, for age-at-onset phenotypes, the model as specified in Equation 1 would not be identifiable because $y_{i}=t_{i}$. Previous studies have proposed to analyze age-specific effects by splitting timescales into non-overlapping intervals. Individuals who have the event in a future interval are treated as right censored, with individuals who have had an event in a previous interval excluded from the analysis. For example, this idea has been suggested by Joshi et al.,^11^ where time intervals of 40–75 and 75+ were used. Although it is correct to conduct the analysis in such a way, doing so requires defining intervals that could be seen as an arbitrary choice, with ill-defined intervals leading to an incomplete understanding of the effect-size distribution. Furthermore, this type of modeling will not scale well with the added number of intervals as each interval requires an additional parameter. Therefore, we propose a form of Cox PH model that allows specifying a functional shape for age-specific effects. To estimate the marginal effect of SNP *j* in chromosome *k*, the general form for CAMP model for each SNP j∈1,…,M is

(Equation 2) $$\lambda_{i}\left(t\right)=\lambda_{0}\left(t\right)\;\exp\;\left(x_{ij}\beta_{j}\left(t\right)+g_{i}^{k}\xi_{j}+z_{i}\delta_{j}\right)\text{,}$$

where $\lambda_{0}\left(t\right)$ is the baseline hazard, *i* denotes the *i*th individual, $x_{ij}$ is the standardized *j*th marker value, $g_{i}^{k}$ is the genetic predictor from all other chromosomes other than the SNP *j* is located at, $\xi_{j}$ is its corresponding effect when estimating marker *j*, $z_{i}$ is the summarized covariate value, and $\delta_{j}$ is its corresponding effect when estimating marker *j*. $\beta_{j}\left(t\right)$ is the effect-size change function for SNP *j* and we define it in two ways. The effect-size function $\beta_{j}\left(t\right)$ should be defined such that its domain is the set of positive real numbers and it is (piecewise) differentiable. Here, we define the effect-size function as a linear function in age

(Equation 3) $$\beta_{j}\left(t\right)=\beta_{j}^{0}+\beta_{j}^{1}\left(t-t_{0}\right)\text{,}$$

where $\beta_{j}^{0}$ is the intercept term, effectively estimating the effect size at time $t_{0}$, and $\beta_{j}^{1}$ is the slope showing how much the effect changes each year. It is possible to define any other parametric shape; for example, the exponential decay function could be a natural choice. However, in the first experiments on real data, the linear effect-size change gave a higher likelihood compared with exponential decay models. Hence, we decided to resort to the linear effect-size model that is easier to interpret, and the variance function can be represented without Taylor expansion-based approximations. Given the effect-size function definition, we can calculate the variance at each time point *t* as

(Equation 4) $$Var\left(\beta_{j}\left(t\right)\right)=Var\left(\beta_{j}^{0}\right)+{\left(t-t_{0}\right)}^{2}Var\left(\beta_{j}^{1}\right)+2\left(t-t_{0}\right)Cov\left(\beta_{j}^{0},\beta_{j}^{1}\right)\text{,}$$

where $Var\left(\beta_{j}^{0}\right)$, $Var\left(\beta_{j}^{1}\right)$, and $Cov\left(\beta_{j}^{0},\beta_{j}^{1}\right)$ can be estimated from the Hessian of the Cox model. We use the linear effect-change definition from Equation 3 to test whether there exists a change in the effect size across the lifespan. As including the genetic values from other chromosomes shares properties with mixed modeling, we are going to refer to this model as the CAMP model.

At each time point *t*, we can define the test statistic function $\chi_{j}^{2}\left(t\right)$ for SNP *j* as the square of the ratio of effect size and the standard error of the effect size

(Equation 5) $$\chi_{j}^{2}\left(t\right)={\left(\frac{{\hat{\beta }}_{j}^{0}+{\hat{\beta }}_{j}^{1}\left(t-t_{0}\right)}{\sqrt{\hat{Var}\left({\hat{\beta }}_{j}^{0}\right)+{\left(t-t_{0}\right)}^{2}\hat{Var}\left({\hat{\beta }}_{j}^{1}\right)+2\left(t-t_{0}\right)\hat{Cov}\left({\hat{\beta }}_{j}^{0},{\hat{\beta }}_{j}^{1}\right)}}\right)}^{2}\text{.}$$

All that remains is to estimate $g_{i}^{k}$, the genetic predictor from all other chromosomes other than the SNP *j*. To do this, we use a BayesW model,^1^ which assumes that for an individual *i*, the age at onset of a disease $y_{i}$ has Weibull distribution, with a re-parameterization of the model to represent the mean and the variance of the logarithm of the phenotype as

(Equation 6) $$E\left(\log\left(y_{i}\right)|\mu ,\beta ,\delta ,\alpha \right)=\mu +\sum_{\varphi =1}^{\Phi }X_{\varphi i}\beta_{\varphi }+Z_{i}\delta \text{,}$$

(Equation 7) $$Var\left(\log\left(y_{i}\right)|\mu ,\beta ,\delta ,\alpha \right)=\frac{\pi^{2}}{6\alpha^{2}}$$

where $X_{\varphi }$ is a standardized genotype matrix containing SNPs allocated to group *φ*, *μ* is an intercept, $\beta_{\varphi }$ is the vector of SNP effects in group *φ*, $Z_{i}$ is additional covariates (such as sex or genetic principal components [PCs]), δ is the additional covariate effect estimates, and *α* is the Weibull shape parameter. For each group, we assume that $\beta_{\varphi }$ are distributed according to a mixture of Gaussian components with mixture-specific proportions $\pi_{\varphi }$ and mixture variances $\sigma_{\varphi 1}^{2},\ldots ,\sigma_{\varphi L}^{2}$ and a Dirac delta at zero, which induces sparsity:

(Equation 8) $$\beta_{\varphi j}∼\pi_{\varphi 0}\delta_{0}+\pi_{\varphi 1}N\left(0,\sigma_{1\varphi }^{2}\right)+\ldots +\pi_{\varphi L}N\left(0,\sigma_{\varphi L}^{2}\right)\text{,}$$

where *L* is the number of mixture components. We estimated the hyperparameters such as genetic variance and prior inclusion probability by grouping markers into “MAF-LD” bins, as recent theory suggests this yields improved estimation.^12^ We used 20 MAF-LD groups that were defined as minor-allele frequency (MAF) quintiles, where we then split each quintile into quartiles by linkage disequilibrium (LD) score. The cutoff points for creating the MAF quintiles were 0.006, 0.013, 0.039, and 0.172; the cutoff points for creating LD score quartiles were 2.11, 3.08, and 4.51 for the first; 3.20, 4.71, and 6.84 for the second; 4.70, 6.89, and 9.94 for the third; 7.65, 11.01, and 15.70 for the fourth; and 10.75, 15.10, and 21.14 for the fifth MAF quintile, exactly as in the age-at-menopause analysis by Ojavee et al.^1^ The posterior mean BayesW model estimates of $\beta_{\varphi }$ are then used to create $g_{i}^{k}$, the genetic predictor from all other chromosomes other than the SNP *j*. This gives a two-step leave-one-chromosome-out (LOCO) approach, where first a BayesW model is used to estimate the genetic predictor and then a marginal age-specific CAMP model is used for the second step. Next, we discuss how we can conduct significance testing in the second step in an efficient manner while ensuring that the type I error is bounded below the fixed threshold *α* even with a more complex model.

### Significance testing

We demonstrate that to limit type I error rate below *α* and given the null hypothesis of no effect at SNP *j* ($\beta_{j}^{0}=0$, $\beta_{j}^{1}=0$), it is sufficient to compare the test statistic $\chi_{j}^{2}\left(t^{*}\right)$ with the $\chi_{df=2}^{2}$ 1−α quantiles at any time point $t^{*}$.

We will naturally assume that $\hat{Var}\left({\hat{\beta }}_{j}^{0}\right)>0$ and $\hat{Var}\left({\hat{\beta }}_{j}^{1}\right)>0$. As $\chi_{j}^{2}\left(t\right)$ is twice differentiable, it is possible to find its respective first and second derivative. This will give us two extreme points $t^{*}$ at which the $\frac{d}{dt}\chi_{j}^{2}\left(t^{*}\right)=0$. Then, the $\chi^{2}$ score has a local maximum if $\frac{d^{2}}{dt^{2}}\chi_{j}^{2}\left(t^{*}\right)<0$ or the $\chi^{2}$ score has a local minimum weakest if $\frac{d^{2}}{dt^{2}}\chi_{j}^{2}\left(t^{*}\right)>0$. It can be shown that the function $\chi_{j}^{2}\left(t\right)$ has two extreme points located at

(Equation 9) $$t_{1}^{*}=-\frac{{\hat{\beta }}_{j}^{0}}{{\hat{\beta }}_{j}^{1}}+t_{0}\text{.}$$

(Equation 10) $$t_{2}^{*}=\frac{{\hat{\beta }}_{j}^{0}\hat{Cov}\left({\hat{\beta }}_{j}^{0},{\hat{\beta }}_{j}^{1}\right)-{\hat{\beta }}_{j}^{1}\hat{Var}\left({\hat{\beta }}_{j}^{0}\right)}{{\hat{\beta }}_{j}^{1}\hat{Cov}\left({\hat{\beta }}_{j}^{0},{\hat{\beta }}_{j}^{1}\right)-{\hat{\beta }}_{j}^{0}\hat{Var}\left({\hat{\beta }}_{j}^{1}\right)}+t_{0}\text{.}$$

Using the second derivative, it can be shown that $\chi_{j}^{2}\left(t\right)$ will always have a (global) maximum at $t_{2}^{*}$ and a (global) minimum ($\chi^{2}\left(t_{1}^{*}\right)=0$) at $t_{1}^{*}$. We find that in the limiting cases, the test statistic is testing the significance of the slope $\lim_{t\to \infty }\chi_{j}^{2}\left(t\right)=\frac{{\left(\beta_{j}^{1}\right)}^{2}}{Var\left(\beta_{j}^{1}\right)}$ and $\lim_{t\to -\infty }\chi_{j}^{2}\left(t\right)=\frac{{\left(\beta_{j}^{1}\right)}^{2}}{Var\left(\beta_{j}^{1}\right)}$. As the domain of $\chi_{j}^{2}\left(t\right)$ is the set of positive real numbers and there are no breakpoints in the function, then $\chi_{j}^{2}\left(t\right)$ is bounded within interval $\left[\chi_{j}^{2}\left(t_{1}^{*}\right),\chi_{j}^{2}\left(t_{2}^{*}\right)\right]$.

We are especially interested in the distribution of the maximum possible $\chi^{2}$ statistic $\chi_{j}^{2}\left(t_{2}^{*}\right)$ under the null hypothesis that both $\beta_{j}^{0}=0$ and $\beta_{j}^{1}=0$.

#### Lemma 1

Under the null hypothesis that both $\beta_{j}^{0}=0$ and $\beta_{j}^{1}=0$, the chi-squared statistic evaluated at the maximum point $t_{2}^{*}$ $\chi_{j}^{2}\left(t_{2}^{*}\right)$ follows a $\chi_{df=2}^{2}$ distribution:

(Equation 11) $$\chi_{j}^{2}\left(t_{2}^{*}\right)∼\chi_{df=2}^{2}\text{.}$$

##### Proof

We define $r:=\frac{\hat{Cov}\left({\hat{\beta }}_{j}^{0},{\hat{\beta }}_{j}^{1}\right)}{\sqrt{\hat{Var}\left({\hat{\beta }}_{j}^{0}\right)\hat{Var}\left({\hat{\beta }}_{j}^{1}\right)}}$ and we express $\chi_{j}^{2}\left(t_{2}^{*}\right)$ such that it would be a sum of two uncorrelated random variables.

(Equation 12) $$\chi_{j}^{2}\left(t_{2}^{*}\right)=\frac{{\left({\hat{\beta }}_{j}^{0}\right)}^{2}\hat{Var}\left({\hat{\beta }}_{j}^{1}\right)-2{\hat{\beta }}_{j}^{0}{\hat{\beta }}_{j}^{1}\hat{Cov}\left({\hat{\beta }}_{j}^{0},{\hat{\beta }}_{j}^{1}\right)+{\left({\hat{\beta }}_{j}^{1}\right)}^{2}\hat{Var}\left({\hat{\beta }}_{j}^{0}\right)}{\hat{Var}\left({\hat{\beta }}_{j}^{0}\right)\hat{Var}\left({\hat{\beta }}_{j}^{1}\right)-\hat{Cov}{\left({\hat{\beta }}_{j}^{0},{\hat{\beta }}_{j}^{1}\right)}^{2}}=\frac{\frac{{\left({\hat{\beta }}_{j}^{0}\right)}^{2}}{\hat{Var}\left({\hat{\beta }}_{j}^{0}\right)}-\frac{2r{\hat{\beta }}_{j}^{0}{\hat{\beta }}_{j}^{1}}{\sqrt{\hat{Var}\left({\hat{\beta }}_{j}^{0}\right)\hat{Var}\left({\hat{\beta }}_{j}^{1}\right)}}+\frac{{\left({\hat{\beta }}_{j}^{1}\right)}^{2}}{\hat{Var}\left({\hat{\beta }}_{j}^{1}\right)}}{1-r^{2}}=\frac{{\left(\frac{{\hat{\beta }}_{j}^{0}}{\sqrt{\hat{Var}\left({\hat{\beta }}_{j}^{0}\right)}}-r\frac{{\hat{\beta }}_{j}^{1}}{\sqrt{\hat{Var}\left({\hat{\beta }}_{j}^{1}\right)}}\right)}^{2}}{1-r^{2}}+\frac{{\left(\sqrt{1-r^{2}}\frac{{\hat{\beta }}_{j}^{1}}{\sqrt{\hat{Var}\left({\hat{\beta }}_{j}^{1}\right)}}\right)}^{2}}{1-r^{2}}\text{.}$$

Under the null hypothesis of $\beta_{j}^{0}=0$ and $\beta_{j}^{1}=0$, we know that $\frac{{\hat{\beta }}_{j}^{0}}{\sqrt{\hat{Var}\left({\hat{\beta }}_{j}^{0}\right)}}∼N\left(0,1\right)$ and $\frac{{\hat{\beta }}_{j}^{1}}{\sqrt{\hat{Var}\left({\hat{\beta }}_{j}^{1}\right)}}∼N\left(0,1\right)$ and therefore

(Equation 13) $$\left(\begin{array}{c} \frac{{\hat{\beta }}_{j}^{0}}{\sqrt{\hat{Var}\left({\hat{\beta }}_{j}^{0}\right)}}-r\frac{{\hat{\beta }}_{j}^{1}}{\sqrt{\hat{Var}\left({\hat{\beta }}_{j}^{1}\right)}}\\ \sqrt{1-r^{2}}\frac{{\hat{\beta }}_{j}^{1}}{\sqrt{\hat{Var}\left({\hat{\beta }}_{j}^{1}\right)}} \end{array}\right)∼N\left(\left(\begin{array}{c} 0\\ 0 \end{array}\right),\left(\begin{array}{cc} 1-r^{2} & 0\\ 0 & 1-r^{2} \end{array}\right)\right)\text{.}$$

The last result implies that under the null of $\beta_{j}^{0}=0$ and $\beta_{j}^{1}=0$, Equation 12 is a sum of two uncorrelated standard Gaussian random variables squared, which means that $\chi_{j}^{2}\left(t_{2}^{*}\right)$ is from the chi-squared distribution with degrees of freedom of 2.

This naturally gives us a rule for hypothesis testing at time $t_{2}^{*}$. If the test fails to disprove the null hypothesis at time $t_{2}^{*}$, it will fail to disprove the null hypothesis at any possible *t*. If the test accepts the alternative hypothesis, it means that there must exist an interval (or at least one point) at which the variable has an effect on the phenotype.

Furthermore, we can show that the quantiles of $\chi_{df=2}^{2}$ distribution result in a stringent-enough test at any time point.

#### Lemma 2

Suppose that we have estimated effect sizes $\beta_{j}^{0}$ and $\beta_{j}^{1}$ from a linear effect change model $\beta_{j}\left(t\right)=\beta_{j}^{0}+\beta_{j}^{1}\left(t-t_{0}\right)$ and that the null hypothesis of $\beta_{j}^{0}=0$ and $\beta_{j}^{1}=0$ holds. Then, for every time point t, the probability of type I error (*α*) is bounded when using the $\chi_{2}^{2}$ distribution 1−α quantile as a critical value.

##### Proof

To prove the lemma, we need to demonstrate that 1−α quantile of $\chi_{2}^{2}$ distribution ($q_{1-\alpha }$) is greater than 1−α quantile of $\chi^{2}\left(t\right)$ at any time point *t* under the null hypothesis $\beta_{j}^{0}=0$ and $\beta_{j}^{1}=0$. Suppose that the maximum test statistic value is achieved at $t_{2}^{*}$ with value $\chi_{j}^{2}\left(t_{2}^{*}\right)$.

We suppose, in contradiction, that under the null hypothesis, there exists some time point $t_{*}\neq t_{2}^{*}$ at which the distribution of $\chi_{j}^{2}\left(t_{*}\right)$ would have a higher 1−α quantile value ${\tilde{q}}_{1-\alpha }$ than the 1−α quantile of $\chi_{df=2}^{2}$ distribution $q_{1-\alpha }$:

$${\tilde{q}}_{1-\alpha }>q_{1-\alpha }\text{.}$$

Given this, we can write the following inequalities,

$$\alpha =P\left(\chi_{j}^{2}\left(t_{*}\right)>{\tilde{q}}_{1-\alpha }\right)<P\left(\chi_{j}^{2}\left(t_{*}\right)>q_{1-\alpha }\right)<P\left(\chi_{j}^{2}\left(t_{2}^{*}\right)>q_{1-\alpha }\right)=\alpha \text{,}$$

where the first inequality follows from the contradiction and the second inequality from the fact that $\chi_{j}^{2}\left(t_{*}\right)$ is the maximum possible value of the test statistic. The inequalities result in a contradiction, which therefore proves the lemma.

An important corollary of this result is that we can use $\chi_{df=2}^{2}$ quantiles to do statistical testing at any time point, and doing tests at (many) different time points will not increase type I error. For example, we can simultaneously test the significance of the slope (corresponds to $\lim_{t\to \infty }\chi_{j}^{2}\left(t\right)$) and significance at $t_{2}^{*}$ (using $\chi_{j}^{2}\left(t_{2}^{*}\right)$) at the 1−α-quantile of $\chi_{df=2}^{2}$ distribution while limiting the type I error at *α* as given the effect and variance estimates.

### UK Biobank data

This project uses UK Biobank data under project 35520. UK Biobank genotypic and phenotypic data are available through a formal request at http://www.ukbiobank.ac.uk. The UK Biobank has ethics approval from the North West Multi-centre Research Ethics Committee (MREC). We first restricted our analysis to a sample of European-ancestry UK Biobank (UKB) individuals. To infer ancestry, we used both self-reported ethnic background (UKB field 21000-0) and genetic ethnicity (UKB field 22006-0) and selected coding 1 in both cases. We projected the 488,377 genotyped participants onto the first two genotypic PCs calculated from 2,504 individuals of the 1,000 Genomes project. Using the obtained PC loadings, we then assigned each participant to the closest 1,000 Genomes project population, selecting individuals with PC1 projection < absolute value 4 and PC2 projection < absolute value 3. Samples were also excluded based on UKB quality control procedures with individuals removed of (1) extreme heterozygosity and missing genotype outliers; (2) a genetically inferred gender that did not match the self-reported gender; (3) putative sex chromosome aneuploidy; (4) exclusion from kinship inference; and (5) withdrawn consent. We used genotype probabilities from version 3 of the imputed autosomal genotype data provided by the UKB to hard call the genotypes for variants with an imputation quality score above 0.3. The hard-call threshold was 0.1, setting the genotypes with probability ≤0.9 as missing. From the good-quality markers (with missingness less than 5% and p value for Hardy-Weinberg test larger than $10^{-6}$, as determined in the set of unrelated Europeans), we selected those with MAF > 0.0002 and rs identifier in the set of European-ancestry participants. We then took the overlap with the Estonian Biobank data described below to give a final set of 8.7 million SNPs using both autosomal chromosomes and the X chromosome. This provides a set of high-quality SNP markers present across both discovery and prediction datasets.

We created the phenotypic data of ANM similarly to Ojavee et al.^1^ We used UKB field 3,581 to obtain the time, if available, and excluded from the analysis (1) women who had reported having and later not having had menopause or vice versa, (2) women who said they had menopause but with no record of the time of menopause (UKB field 2,724), (3) women who have had a hysterectomy or the information about this is missing (UKB field 3,591), and (4) women whose menopause is before age 33 or after 65. Within the UKB data, there were a total of 173,424 unrelated (only one person kept from second-degree or closer relative pairs) European-ancestry women, out of which 125,697 had experienced menopause and 47,727 had not had menopause based on data field 2,724. For computational convenience when conducting the joint BayesW analysis, we created an additional subset of markers by removing markers in very high LD through the selection of the highest MAF marker from any set of markers with LD $R^{2}\geq 0.8$ within a 1-Mb window. These filters resulted in a dataset with 173,424 individuals and 2,174,071 markers for the first-step estimation of the LOCO genetic predictors, and then in the second step CAMP model we analyzed 8.7 million SNPs using both autosomal chromosomes and the X chromosome.

### Estonian Biobank data

To replicate the findings, we used the Estonian Biobank with 70,082 women (22,740 with menopause and 47,342 without menopause). For access to be granted to the Estonian Biobank genotypic and corresponding phenotypic data, a preliminary application must be presented to the oversight committee, who must first approve the project. Ethics permission must then be obtained from the Estonian Committee on Bioethics and Human Research, and finally a full project must be submitted and approved by the Estonian Biobank. This project was granted ethics approval by the Estonian Committee on Bioethics and Human Research (https://genomics.ut.ee/en/content/estonian-biobank). Similar to the UKB data, we used the age range of 33–65 and thus excluded women with age of menopause outside this interval. In the total Estonian Biobank data, there were 195,432 individuals genotyped on the Illumina Global Screening Array (GSA), which were imputed to an Estonian reference created from the whole-genome sequence data of 2,244 participants.^13^ From 11,130,313 markers with imputation quality scores >0.3, we selected SNPs that overlapped with those selected in the UKB as described above by using the same SNP sets for the first and second steps of the analyses.

### Analysis of ANM

We investigated the three questions as proposed in Figure 1: (1) we checked the interval at which there is a significant effect, (2) we tested the significance at ages at which the variants had the most evidence for an effect, and (3) we tested for the existence of an age-specific effect.

### Testing the interval at which a variant is significant

**Figure 2:**
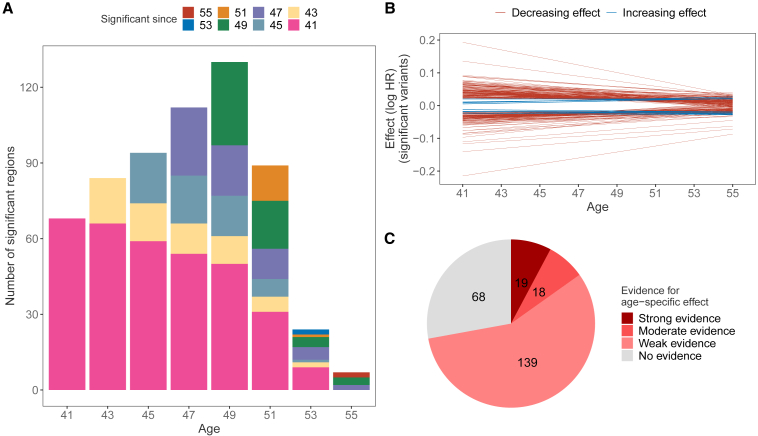
Age distribution of significant effects and effect-size change (A) We evaluated the effect-size and standard error estimates for every SNP at each age on a grid from 41 to 55 and counted the number of significant hits replicated in the Estonian Biobank. The significant focal hits were mapped to consecutive ages, summarizing the count since when the effects were significant. (B) Effect sizes (log hazard ratio, HR) for 245 significant effects; the majority of variants have a larger absolute effect size at age 41 than at age 55, and only 9 variants have an increasing effect size. We observe that the model manages to capture the effect-size change for many variants. (C) Classification of the menopause-associated variants by age-specific evidence by testing whether the slope parameter is equal to zero. Variants with weak evidence have a p value lower than p<0.05, moderate evidence requires $p<5\times 10^{-8}$, and strong evidence requires the slope to be significant also in the replication dataset.

We evaluated the test statistic function using Equation 5 at ages 41, 43, 45, 47, 49, 51, 53, and 55 and calculated the p values using the $\chi_{df=2}^{2}$ distribution quantiles, as suggested by our theory above. On these results, at each age, we applied the clumping procedure (using plink 1.9^14^) with a window size of 1 Mb, LD threshold of $r^{2}=0.05$, p value threshold for index SNPs of $p=5\times 10^{-8}$, and no p value threshold for other SNPs belonging to a clump of an index SNP. To detect clumps with an independent signal, we applied the COJO procedure^15^ implemented in GCTA software^16^ with a window size of 1Mb, and the SNPs were considered independent if the p value in the joint model was less than $5\times 10^{-8}$. The independent index SNPs from the COJO analysis were then replicated at each age in the Estonian Biobank data with replication defined as a p value lower than 0.05 and the same effect-size estimate sign as in the discovery analysis. To check the period during which a SNP has a significant effect (Figure 2A), we checked whether the same SNP or a SNP in the same clump also has an effect in the consecutive grid point. Specifically, we took all the significant independent and replicated SNPs at ages 41 and 43 and then checked whether the index SNPs of age 41 mapped directly to an index SNP at age 43 or a clump of an index SNP. Then, we compared the ages 43 and 45 and iteratively so forth until 55.

### Testing the age with the maximum effect evidence

We tested the significance at ages at which the variants had the most evidence for an effect to understand the total number of significant effects and possibly identify previously undiscovered loci. Furthermore, we defined a period of interest between ages 45 and 52 so the significance would only be evaluated during this period. That was done to avoid over-interpretation of the linear effect at uncommon high or low ages. Therefore, using Equation 10, we first calculated the age $t_{2}^{*}$ at which the test statistic achieves the highest value, and secondly, we evaluated the chi-squared function (Equation 5) at that age $t_{2}^{*}$. If the time point $t_{2}^{*}$ was outside of the interval (45,52), then we instead evaluated the function at the time point where the function $\chi_{j}^{2}\left(t\right)$ achieved its maximum within the interval (either age 45 or 52). We compared the chi-squared statistics with the $\chi_{df=2}^{2}$ distribution to calculate the p values. We applied a similar procedure to the previous case. First, we applied the clumping procedure (using plink 1.9^14^) with a window size of 1 Mb, LD threshold of $r^{2}=0.05$, p value threshold for index SNPs of $p<5\times 10^{-8}$, and no p value threshold for other SNPs belonging to a clump of an index SNP. Secondly, to detect clumps with an independent signal, we applied the COJO procedure^15^ implemented in GCTA software^16^ with a window size of 1 Mb, and the SNPs were considered independent if the p value in the joint model was less than $5\times 10^{-8}$. We then checked the independent and significant SNPs from the COJO analysis for previous association signals. We removed all the markers that had a correlation of $r^{2}>0.1$ with a marker that had been previously found associated with ANM using the GWAS catalog (published until April 2022) and LDtrait tool with the British in England and Scotland population. Furthermore, we specifically compared our candidate set for the significant SNPs reported by Ruth et al.,^9^ removing the markers reported by them and those with a correlation $r^{2}>0.1$. Finally, we checked our candidate set with the Phenoscanner database^17^^,^^18^ to find any previous associations with variants of interest or variants in LD. The index SNPs not removed by the three filters were then replicated at age with maximum evidence in the period (45,52) in the Estonian Biobank data, with replication defined as a p value lower than 0.05 and the same effect estimate sign as in the discovery analysis.

### Testing for an age-specific effect

To verify whether there is a genome-wide significant age-specific effect for every SNP *j*, we checked whether the slope parameter is significantly different from 0

$$H_{0}:\beta_{j}^{1}=0\text{,}$$

where $\beta_{j}^{1}$ is estimated from the model specified in Equation 2. The chi-squared statistic was calculated as the squared ratio of the slope size estimate and standard error estimate. As this quantity naturally corresponds to $\lim_{t\to \infty }\chi_{j}^{2}\left(t\right)$ where $\chi_{j}^{2}\left(t\right)$ is defined as in Equation 5, we compare the test statistic again with the $\chi_{df=2}^{2}$ distribution quantiles to get the p values. Similarly to two previous cases, we first applied the clumping procedure (using plink 1.9^14^) with a window size of 1 Mb, LD threshold of $r^{2}=0.05$, p value threshold for index SNPs of $p<5\times 10^{-8}$, and no p value threshold for other SNPs belonging to a clump of an index SNP. Second, to detect clumps with an independent signal, we applied the COJO procedure^15^ implemented in GCTA software^16^ with a window size of 1 Mb, and the SNPs were considered independent if the p value in the joint model was less than $5\times 10^{-8}$. The independent index SNPs from the COJO analysis were then replicated in the Estonian Biobank data, with replication defined as a p value lower than 0.05 and the sign of the slope the same as in the discovery analysis. We classified the SNPs with different levels of evidence of age-specific effects. SNPs with a p value below the nominal significance threshold (p<0.05) are said to have at least weak evidence for an age-specific effect; variants with a p value below the genome-wide significance threshold ($p<5\times 10^{-8}$) are said to have at least moderate evidence for an age-specific effect; variants with moderate evidence that also replicate in the Estonian Biobank are considered to have strong evidence for an age-specific effect. The variants that do not fall under these three categories are said to have no evidence for age-specific effects.

### Enrichment analysis

We used recently presented Downstreamer software^19^ to identify genes connected to our association study results through gene expression and to identify enriched pathways. We calculated test statistics using Equation 5 at ages 41, 43, 45, 47, 49, 51, 53, and 55 and calculated the p values using the $\chi_{df=2}^{2}$ distribution quantiles, as suggested by our theory above.

Downstreamer implements a strategy that accounts for LD structure and chromosomal organization, operating in two steps. In the first step, gene-level prioritization scores were calculated for each age group’s summary statistics and a null distribution. This aggregates p values per variant into a p value per gene while accounting for local LD structure. GWAS gene p values were calculated for all 20,327 protein-coding genes (Ensembl release version 75). The gene p values were then converted to gene *Z* scores for use in subsequent analysis. To account for the long-range effects of haplotype structure, which results in genes getting similar gene *Z* scores, a generalized least-squares (GLS) regression model is used, which requires a gene-gene correlation matrix. This correlation matrix is calculated by first simulating 10,000 random phenotypes by drawing phenotypes from a normal distribution and then associating them to the genotypes of the 1000 Genomes phase 3 non-Finnish European samples. The GWAS gene *Z* scores for each of the 10,000 simulated GWAS signals alongside the Pearson correlations between the GWAS gene *Z* scores are then calculated. Correlations between simulated GWAS gene *Z* scores reflect the underlying LD patterns and chromosomal organization of genes. An additional 10,000 GWASs were simulated to empirically determine enrichment p values, and, finally, an additional 100 simulations were used to estimate the false discovery rate (FDR).

In the second step, the gene-level prioritization scores were associated with the co-regulation matrix and pathway annotations. We used a previously generated co-regulation matrix that is based on a large multi-tissue gene network.^19^ Publicly available RNA-seq samples were downloaded from the European Nucleotide Archive (https://www.ebi.ac.uk/ena) containing 56,435 genes and 31,499 samples covering a wide range of human cell types and tissues. 165 leading PCs representing 50% of the variation were selected. For protein-coding genes, centered and scaled eigenvectors for these 165 components (mean = 0, SD = 1) were calculated. The co-regulation matrix is then defined as the Pearson correlation between the genes from the scaled eigenvector matrix, with diagonal zero and Pearson r values converted to *Z* scores. To identify pathway and disease enrichments, the Human Phenotype Ontology (HPO), KEGG, Reactome and Gene Ontology (GO) Biological Process, Cellular Component, and Molecular Function databases were used. A *Z* score per pathway or term per gene is calculated, giving how much each gene contributes to these gene sets. We collapsed correlated genes in parallel with the GWAS step to ensure compatibility with the GWAS gene *Z* score and scaled all pathway *Z* scores to zero mean and unit variance.

Real and simulated GWAS *Z* scores were rank-based inverse-normal transformed. A linear model was used to correct for gene length, as longer genes will typically harbor more SNPs. Genes with a Pearson correlation r≥0.8 in the 10,000 GWAS permutations were treated as one gene. A GLS regression is used to associate the GWAS gene *Z* scores to the pathway *Z* scores and co-regulation *Z* scores, with $\beta ={\left(X^{T}\omega^{-1}X\right)}^{-1}X^{T}\omega^{-1}y$, where *β* is the estimated effect size of a pathway, term, or gene from the co-regulation matrix; *ω* is the gene-gene correlation matrix; X is the design matrix of real GWAS *Z* scores; and y is the vector of gene *Z* scores per pathway, term, or gene from the co-regulation matrix. *β* for the 10,000 random GWASs were estimated in the same way and used to estimate the empirical p value for *β*. These two analyses result in pathway enrichments and core gene prioritizations, respectively. The gene-gene correlation matrix derived from the 10,000 permutations is used as a measure of conditional covariance of the error term (*ω*) in the GLS to account for the relationships between genes due to LD and proximity. We present full results for each age in Table S2. Pathway enrichment analysis for age 41, Table S3. Pathway enrichment analysis for age 43, Table S4. Pathway enrichment analysis for age 45, Table S5. Pathway enrichment analysis for age 47, Table S6. Pathway enrichment analysis for age 49, Table S7. Pathway enrichment analysis for age 51, Table S8. Pathway enrichment analysis for age 53, Table S9. Pathway enrichment analysis for age 55. In the main figures, we present the results significant at both FDR correction and Bonferroni correction offered by the Downstreamer software.

### Genetic correlations

We used LD score regression to calculate genetic correlations among the test statistics generated using Equation 5 at ages 41, 43, 45, 47, 49, 51, 53, 55, and among these ages and other phenotypes using publicly available GWAS summary data. We used UKB results for 100 phenotypes released by Neale group and the Global Biobank Meta-analysis Initiative consortium. The significance threshold of 0.05 was corrected by the total number of tests (800). We present only estimates in Figure 4 where the correlation was significant in at least one age point.

### Mendelian randomization

We calculated the causal effect estimates that ANM at different ages has on various traits (Table S11) using Mendelian randomization (MR), a statistical method that utilizes the randomized inheritance of genetic variations in the population to estimate the potential causal effect a modifiable risk factor or exposure has on a health-related outcome of interest.^20^^,^^21^ The genetic variants used as instrumental variables (IVs) for our exposure were selected to have a genome-wide significant association with the exposure ($p<5\times 10^{-8}$) and were then pruned using LD distance to ensure that they were independent. This was done using the “ld_clump” function of the “ieugwasr” R package^22^ with default settings (clump_kb = 10,000, clump_r2 = 0.001, clump_p = 0.99, pop = “EUR”). After the IVs of our exposure were selected, their association effects were then obtained for each of our outcome traits of interest. A single-sided t test was carried out to check whether the IVs had a stronger association with the outcome than with the exposure and were subsequently removed if so (for violating the MR assumptions). The two sets of association effects were then harmonized and used to calculate the causal effect estimates using the inverse-variance weighted method found in the “TwoSampleMR” R package.^23^ This analysis was repeated for each varying age of our exposure. It is important to note that in the case of educational attainment as an outcome, there were few exposure IVs that overlapped with the outcome genetic variants, especially as the age increased; hence, in the presence of a single IV, a Wald ratio was used to calculate the MR causal effect estimate. Moreover, when the trait was of a case-control nature, the effective sample size was calculated using the following formula: (4^∗^cases^∗^controls)/(cases+controls).^24^

## Results

### Modeling effect-size change reveals previously unreported loci

We show in the methods that CAMP determines the time dependency of marker age-at-onset associations without a significant multiple-testing burden. We applied the CAMP model to 173,424 unrelated observations of self-reported ANM in the UKB data (125,697 reported events and 47,727 right-censored observations; 8,747,951 SNPs) and 70,082 observations in the Estonian Biobank (22,740 reported events and 47,342 censored observations, Figure S1). We find 312 ANM associations in the UKB, of which 226 replicate previous studies^7^^,^^8^^,^^9^ and 19 are previously unreported and replicate for the first time within the Estonian Biobank (Table 1; Figures 1B and S2). In addition, we find 67 associations that have not previously been reported, but they did not replicate in the Estonian Biobank. Nevertheless, 46 out of 67 previously unreported associations show consistency with signs in the discovery and replication datasets (Fisher’s exact test, p=0.007), suggesting that a larger replication dataset could lead to further replications. To test for novelty, we test for significance at time points where the evidence is the highest within the intervals 45 to 52 (Figure 1A, see methods). For significant SNPs detected in the UKB, the age distribution of maximum association evidence is concentrated between the ages of 43 and 51 (Figure S3). That is different compared with the maximum association evidence age distribution for all SNPs, which has thicker tails with nearly four times higher standard deviation even if the distributions have similar centers (median age of 51 and 49 for all SNPs and significant SNPs, respectively) (Figure S3). In conclusion, the CAMP approach yields an increase in previously unreported loci: 8% of the replicated marker associations are previously unreported, a somewhat expected increase resulting from our approach leveraging potentially existing age specificity.

**Table 1:** Previously undiscovered regions affecting age at menopause

**SNP**	**Chr**	**Position**	**Eff/oth**	**MAF**	**Nearest gene**	**Maximum effect**	**Age at maximum**	**p value**	**Effect at 49**	**Yearly effect change**
rs16852403	1	178,039,226	C/T	0.200	RASAL2-AS1^∗^	0.026	45.0	$5.70\times 10^{-9}$	0.014	−0.0032
rs60897342	1	235,512,110	T/C	0.475	GGPS1^∗^	−0.018	49.6	${4.32\times 10}^{-10}$	−0.018	0.0008
rs77629370	2	27,251,504	T/C	0.062	MAPRE3^∗^	0.023	48.7	$3.52\times 10^{-14}$	0.022	−0.0017
rs6544660	2	43,688,496	C/T	0.451	THADA	−0.021	48.8	$7.22\times 10^{-12}$	−0.020	0.0016
rs16839858	2	204,366,776	A/G	0.166	RAPH1	−0.017	51.0	$1.30\times 10^{-9}$	−0.016	−0.0005
rs6443930	3	183,754,294	C/G	0.439	HTR3D	0.022	50.1	$2.15\times 10^{-14}$	0.022	−0.0004
rs816734	5	154,272,947	T/C	0.350	GEMIN5	0.021	47.9	$1.26\times 10^{-10}$	0.019	−0.0022
rs142490551	5	176,369,037	C/G	0.019	UIMC1	0.021	49.9	$3.31\times 10^{-14}$	0.021	−0.0003
rs191306205	6	31,739,684	T/C	0.025	VWA7	0.020	48.5	$1.36\times 10^{-11}$	0.020	−0.0016
rs60375899	7	860,846	A/G	0.130	SUN1	0.020	49.5	$3.93\times 10^{-12}$	0.020	−0.0009
rs2905065	9	136,958,528	C/T	0.326	RP11-349K21.1^∗^	0.027	48.0	$1.65\times 10^{-16}$	0.024	−0.0026
rs7946546	11	63,595,648	G/A	0.473	C11orf84^∗^	0.018	51.8	$3.32\times 10^{-9}$	0.014	0.0012
rs17180987	12	66,844,882	A/G	0.018	GRIP1	−0.021	47.8	$3.60\times 10^{-9}$	−0.018	0.0024
rs1285841	14	91,881,387	T/C	0.443	CCDC88C	−0.022	47.4	$3.36\times 10^{-10}$	−0.018	0.0024
rs143569302	15	41,258,121	T/C	0.040	CHAC1^∗^	0.021	49.4	$1.37\times 10^{-13}$	0.022	−0.0009
rs11638671	15	63,795,628	C/T	0.344	USP3^∗^	−0.031	45.0	$2.10\times 10^{-11}$	−0.015	0.0039
rs11647700	16	12,108,743	T/C	0.073	SNX29	0.020	50.3	$9.58\times 10^{-13}$	0.020	0.0000
rs9807043	17	48,875,077	T/C	0.158	RP11-294J22.5^∗^	0.017	49.5	$1.65\times 10^{-9}$	0.018	−0.0008
rs118159243	17	62,633,136	C/G	0.017	SMURF2	0.020	47.7	$6.99\times 10^{-10}$	0.017	−0.0019

For 8.7M SNPs, we determined the age at which there is the strongest evidence for an effect within the CAMP model is in age range 45–52. Then, given the age identified for each SNP, we tested for significance at this age by using the CAMP model results, and we obtained effect-size and standard error estimates. The results were then LD clumped such that the index SNPs would have a p value below $5\times 10^{-8}$, and SNPs could be added to a clump if they were 1 Mb from the index SNP, were correlated with $r^{2}>0.05$, and were nominally significant (p<0.05). We then used the COJO method from the GCTA software (see methods) to find clumps with independent signals by conducting a stepwise selection of index SNPs in a 1-Mb window, and we considered SNPs independent if they had a p value below $5\times 10^{-8}$ in the joint model. To determine novelty, we then removed all the markers that had a correlation of $r^{2}>0.1$ with a marker that had been previously found associated with age at menopause using the GWAS catalog and LDtrait tool with the British in England and Scotland population. For the remaining SNPs, we conducted an additional literature review using the Phenoscanner database (see methods) to find any previous associations with variants of interest or variants in LD. The remaining candidates for previously unreported associations were then tested in the Estonian Biobank. Replication was defined as a p value lower than 0.05 and the direction of the effect size same in both the original analysis and the replication analysis. The effect-size estimates are reported on the log hazard scale. The column named “nearest gene” is mapped from the SNP using ANNOVAR software (see methods), with ∗ in that column denoting intergenic regions.

### Proportion and change of age-specific effects

For quantifying the existence of age-specific effects, we first test the null hypothesis of whether the slope term $\beta_{j}^{1}$ is different from 0, with a rejection of the null implying the existence of a time-varying genetic effect. Of the 245 UKB associations (226 previously reported + 19 previously unreported associations), we find that 72% (176) show at least nominally significant (p<0.05) age-specific effects within UK women (Figure 2C). 37 of these 176 associations, representing 15% of all associations, have a slope with a genome-wide significant p value ($p<5\times 10^{-8}$), constituting a more stringent criterion (strong and moderate evidence in Figure 2C). Checking the significance of the slope for all SNPs (not only genome-wide significantly associated to ANM), we find 63 regions that exhibit a significant slope term in the UKB (Tables 2 and S1), and we replicate the age-specific effects for 20 regions in the Estonian Biobank (Table 2), yielding a replication rate of 32% (Figure 2C). These 20 variants have stronger effect sizes earlier in life that mostly decay toward zero after age 50 (Figure S5), making them early-ANM specific. Although 139 variants do not pass the threshold for genome-wide significance, they still indicate that for many regions previously identified as menopause associated, the assumption of constant effect size (assumption of proportional hazards at the SNP) is generally invalid. Indeed, the 43 UKB-discovered variants with significant slope terms that did not replicate in the Estonian Biobank had effect-size directions that were broadly concordant across studies (Fisher’s exact test p=0.051, Table S1).

**Table 2:** Regions with a genome-wide significant age-specific effect on age at natural menopause replicated in the Estonian Biobank

**SNP**	**Chr**	**Position**	**Eff/oth**	**MAF**	**Nearest gene**	**Yearly effect change**	**Slope p value**	**Strongest evidence p value**
rs6684319	1	39,334,988	A/G	0.310	MYCBP	0.0061	$1.12\times 10^{-19}$	$9.63\times 10^{-57}$
rs185012833	2	62,779,457	C/A	0.003	PSAT1P2^∗^	−0.0033	$6.03\times 10^{-9}$	$1.81\times 10^{-6}$
rs6760293	2	171,816,531	A/T	0.373	GORASP2	−0.0040	$1.36\times 10^{-9}$	$7.33\times 10^{-34}$
rs12503643	4	185,746,088	T/G	0.399	ACSL1	0.0049	$3.05\times 10^{-13}$	$4.28\times 10^{-61}$
rs274722	5	6,718,668	T/C	0.406	PAPD7	−0.0040	$1.06\times 10^{-9}$	$3.64\times 10^{-25}$
rs58400555	5	176,454,081	T/A	0.484	ZNF346	0.0039	$5.34\times 10^{-9}$	$3.21\times 10^{-179}$
rs2077491	6	31,606,376	C/T	0.473	BAG6^∗^	0.0053	$1.67\times 10^{-15}$	$1.06\times 10^{-60}$
rs728900	10	131,590,300	A/T	0.420	RP11-109A6.3^∗^	−0.0050	$5.79\times 10^{-14}$	$4.19\times 10^{-28}$
rs75770066	12	66,704,225	G/A	0.033	HELB	0.0049	$6.62\times 10^{-12}$	$1.58\times 10^{-212}$
rs11638671	15	63,795,628	C/T	0.344	USP3^∗^	0.0039	$3.62\times 10^{-9}$	$1.09\times 10^{-10}$
rs33650	16	11,978,769	C/T	0.385	GSPT1	0.0042	$3.28\times 10^{-10}$	$6.75\times 10^{-65}$
rs1433753	16	34,879,951	T/C	0.441	RP11-14K3.1^∗^	−0.0048	$4.21\times 10^{-13}$	$2.26\times 10^{-23}$
rs8071278	17	41,193,910	T/A	0.335	BRCA1^∗^	0.0048	$5.01\times 10^{-13}$	$4.70\times 10^{-50}$
rs1991401	17	62,502,435	G/A	0.310	DDX5	−0.0048	$2.51\times 10^{-13}$	$2.96\times 10^{-36}$
rs16960290	19	55,799,918	T/C	0.433	BRSK1	0.0079	$2.80\times 10^{-32}$	$1.11\times 10^{-86}$
rs117146677	19	55,833,868	A/G	0.009	TMEM150B	−0.0053	$1.50\times 10^{-18}$	$1.61\times 10^{-43}$
rs299163	19	56,321,414	C/A	0.066	NLRP11	0.0042	$4.26\times 10^{-10}$	$8.36\times 10^{-17}$
rs8124538	20	61,300,863	A/G	0.212	SLCO4A1	−0.0039	$1.65\times 10^{-9}$	$3.63\times 10^{-54}$
rs6631137	X	30,665,762	C/T	0.316	GK^∗^	−0.0064	$1.60\times 10^{-22}$	$5.43\times 10^{-43}$
rs67596711	X	152,638,744	G/T	0.499	ZNF275^∗^	0.0055	$1.28\times 10^{-16}$	$3.82\times 10^{-26}$

For each SNP, we tested the significance of the slope parameter using the CAMP model. The results were then LD clumped such that the index SNPs would have a p value below $5\times 10^{-8}$, and SNPs could be added to a clump if they were 1 Mb from the index SNP, were correlated with $r^{2}>0.05$, and were nominally significant (p<0.05). We then used the COJO method from the GCTA software (see methods) to find clumps with independent signals by conducting a stepwise selection of index SNPs in a 1-Mb window, and we considered SNPs independent if they had a p value below $5\times 10^{-8}$ in the joint model. The candidates for significant slope were then replicated in the Estonian Biobank. Replication was defined as a p value lower than 0.05 and the direction of the effect size same in both the original analysis and the replication analysis. The effect-size estimates are reported on the log hazard scale. The column named “nearest gene” is mapped from the SNP with ANNOVAR software (see methods); an asterisk (^∗^) in that column denotes intergenic regions; chromosome X’s nearest gene was determined by using the UCSC Genome Browser. The column named “strongest evidence p value” indicates the p value at the age when there is the strongest evidence for an effect.

Second, we observe that the number of regions affecting ANM changes considerably with the peak number of ANM-affecting regions observed at age 49 (Figure 2A). Moreover, we find that the period during which a particular region can significantly impact ANM varies considerably with only half of the significant associations at age 47 also significant at age 41. In general, we observe that effects tend to become insignificant with increasing age, with the drop in significance occurring at age 53, so that by age 55 only 8 loci have a genome-wide significant effect on ANM (Figure 2A). A similar result can be seen if we observe the distribution of ages when the evidence for the menopause effect is the strongest (Figure S3), as very few significant SNPs achieve the strongest association after age 51.

In contrast with most associations discovered at age 49, the general trend across 245 significant SNPs is that the effect size estimates shrink toward zero (Figure 2B). That might imply that the increase in the number of discoveries in the period 41 to 49 is instead due to the reduction in the standard error, and with a higher sample size, it could be possible to detect more associations already at earlier ages. Interestingly, only 9 of the significant SNPs have a larger absolute effect size at age 55 than at 41. That is in line with many previous results reporting a reduction in relative genetic risks with the increase in age.^25^^,^^26^ Finally, we observe that there exists a stark difference between the effect-size profiles of significant and insignificant effects (Figure S4) with a much narrower effect-size distribution for the non-significant SNPs. Meanwhile, menopause-associated variants stand out as their effect size can change greatly across the period of interest. We find it important to stress that the interpretation of the effects is done using (log−) hazard ratios as the underlying model is the CAMP model. Specifically, in the context of the CAMP model, hazard ratios need to be interpreted at each age separately as the hazard ratio changes with age, for example as seen in Figure 2.

Our analysis differs in one other key way from previous ANM genetic association studies. Here, we do not censor women who were placed on hormone replacement therapy (HRT). In survival models, declaring HRT individuals as censored makes the modeling assumption that age-at-HRT start and ANM are independent. They are clearly not, as for women on HRT there is a correlation between the age of HRT and ANM of 0.58 within the UKB. For women who were placed on HRT prior to the recorded date of ANM, this correlation is stronger at 0.69. A Cox Proportional Hazards model for ANM including a categorical covariate of whether a woman was given HRT prior to menopause (1 if on HRT prior to menopause, 0 otherwise) shows that censoring for HRT prior to ANM would significantly censor for earlier menopause (HR = 0.95, $p=6.08\times 10^{-15}$). Thus, censoring for HRT is not the optimal modeling choice, and additionally, it results in the loss of 34,031 observations, reducing power. Nevertheless, we conduct a sensitivity analysis of our estimated effect sizes with and without adding HRT as a time-varying covariate to the CAMP model at the 312 top loci (both replicated and unreplicated regions) identified within our study (Figure S6). We find very strong concordance of effect sizes across loci (Figure S6), highlighting that in practice these different modeling choices have no detectable impact on the leading SNP association findings.

Finally, we highlight notable examples of the previously unreported replicated associations with significant slope terms. For example, we note that chr11: 63,595,648 (in GRCh37 coordinates), which is downstream of *SPINDOC*, is increasingly associated with menopause genetic risk as age increases, with the highest effect size being at later age groups. Another example is chr15: 63,795,628, which is upstream of *USP3*, where the menopause association disappears with increasing age. Both of these associations were previously suggestively associated with ANM, but they pass the significance threshold in the UKB, and they replicate in the Estonian Biobank, when our proposed model was used.

### Properties of previously unreported and age-specific genetic associations

**Figure 3:**
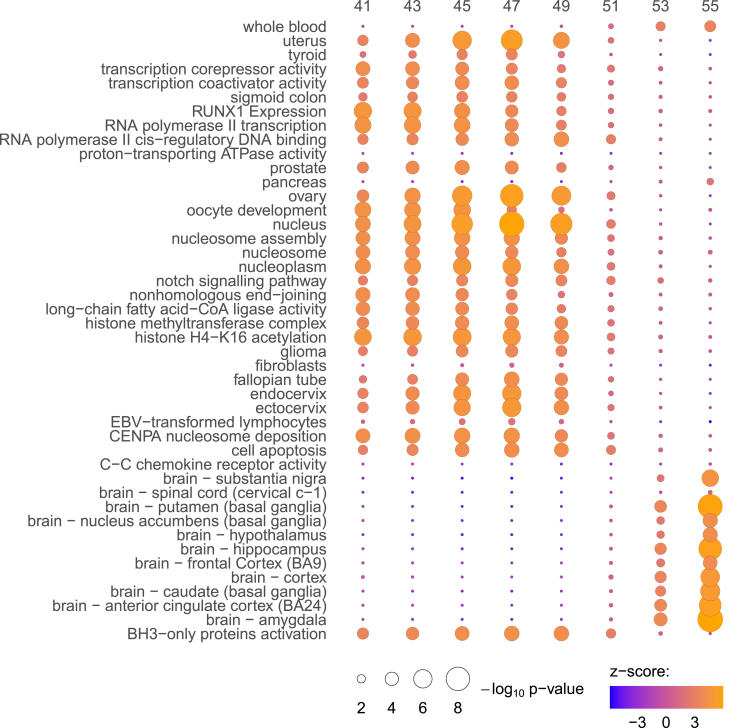
Age-specific enrichment of genetic associations across multiple genomics resources We evaluated the significance of every SNP at each age on a grid from 41 to 55 and from the resulting summary statistics we tested for enrichment across multiple genomics resources. Circle circumference gives the $-\log_{10}$ p value and the color gives the enrichment *Z* score calculated from the Downstreamer software. GTEx tissue-specific expression and GO terms are given on the y axis for annotations with genome-wide significance after multiple testing correction at one or more age groups. Full results are given in Table S2. Pathway enrichment analysis for age 41, Table S3. Pathway enrichment analysis for age 43, Table S4. Pathway enrichment analysis for age 45, Table S5. Pathway enrichment analysis for age 47, Table S6. Pathway enrichment analysis for age 49, Table S7. Pathway enrichment analysis for age 51, Table S8. Pathway enrichment analysis for age 53, Table S9. Pathway enrichment analysis for age 55.

We conduct a number of follow-up analyses to support our age-specific association results. First, we test for significant enrichment of the summary statistics generated by our approach for each age group. For all categories showing significant enrichment after Bonferroni multiple testing correction, we find that their significance does not hold across all age groups (Figure 3; Table S2. Pathway enrichment analysis for age 41, Table S3. Pathway enrichment analysis for age 43, Table S4. Pathway enrichment analysis for age 45, Table S5. Pathway enrichment analysis for age 47, Table S6. Pathway enrichment analysis for age 49, Table S7. Pathway enrichment analysis for age 51, Table S8. Pathway enrichment analysis for age 53, Table S9. Pathway enrichment analysis for age 55).

Effect sizes for ANM between the ages of 41–49 were enriched in genes differentially expressed in the uterus, thyroid, prostate, ovary, fallopian tubes, and cervix within the Genotype-Tissue Expression (GTEx) consortium data (Figure 3). Additionally, we find enrichment between the ages of 41 and 49 for KEGG pathway NOTCH signaling associated with cell proliferation and death, the GO terms for an intrinsic pathway for apoptosis, and BH3-only proteins (Figure 3). In contrast, associations with variation in ANM for individuals older than 51 were all enriched for genes with differential expression in several brain regions within the GTEx data with no evidence for enrichment in reproductive tissues (Figure 3). These results suggest that genetic effects may differ across the age range.

**Figure 4:**
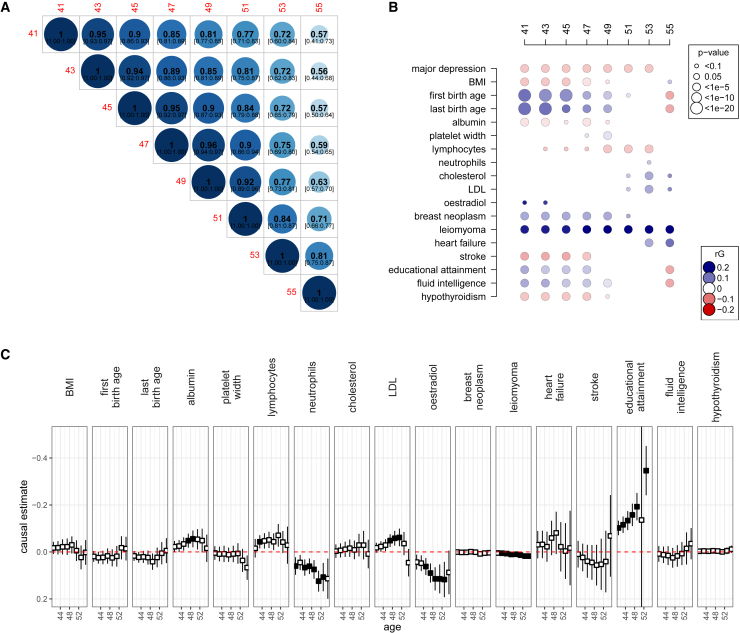
Age-specific genetic correlations and causality of ANM and health-related outcomes (A and B) We evaluated the effect-size and standard error estimates for every SNP at each age on a grid from 41 to 55, and from the resulting summary statistics we tested for genetic correlations among (A) age groups and (B) among observed ANM values at the age groups and 100 other health-related indicators and outcomes using LD score regression. In (B), we present correlations for outcomes with evidence of a significant non-zero genetic correlation at one age group or more. (C) In (C), we present results from inverse-weighted Mendelian randomization that estimates the potential causal relationship between ANM and outcomes where a significant genetic correlation was found in (B) across ages. Black boxes depict ages for which significant causal estimates were found. Major depression is excluded from (C) as there are no instrumental variables available for this analysis. Full results from a range of Mendelian randomization models are given in Table S10.

Our next follow-up analysis used LD score regression, where we find that genetic correlations across ages are significantly less than 1 (Figure 4A). Genetic correlations of ANM and other phenotypes were also largely age dependent (Figure 4B). Note here that effect-size estimates for ANM are calculated on the menopause hazard scale, and thus a positive correlation estimated by LD score regression would refer to a high hazard of ANM (earlier ANM) corresponding to high trait values; in other words, the observed value of ANM and the trait are in fact negatively correlated. Thus, to ease interpretation, we flip the sign of the estimated correlation to display the genetic correlation of the observed values of ANM and each trait.

Between 41 and 49 years, we find a significant positive genetic correlation of observed ANM values with age at last birth and age at first birth (Figure 4B), implying a genetic relationship between later reproduction and later ANM within this age range.

Of significant note, genetic propensity for breast cancer was significantly associated with a later ANM (Figure 4B) before age 51. This supports previous evidence, where genetically mediated delays in ANM were found to increase the relative risks of several hormone-sensitive cancers.^8^ Additionally, evidence linking exposure to high levels of estrogen hormones with an increased risk of breast cancer is supported by a significant positive genetic correlation of ANM and oestradiol levels for women of early menopause, implying a genetic propensity for high estrogen levels associated with a genetic propensity for a later ANM (Figure 4B). Furthermore, we find that a high genetic risk for leiomyoma is consistently associated with later ANM (Figure 4B). Together with our enrichment results presented above (Figure 3) showing early ANM genetic associations are enriched for genes differentially expressed in female reproductive organs, oocytes, and DNA damage-repair mechanisms, our findings suggest that at the genetic level, breast cancer risk, hormone levels, and ANM are linked prior to age 50.

We also find significant genetic correlations implying that genetically mediated later ANM is correlated with a lower genetic predisposition to hypothyroidism, stroke, major depression, blood albumin levels, and obesity prior to age 51 (Figure 4B). For women at later ages, we find positive genetic correlations of ANM with cholesterol, low-density lipoprotein (LDL), obesity, and heart failure, implying that later ANM is correlated with an increased genetic predisposition for these metabolic-associated health measures (Figure 4B).

We find a significant positive genetic correlation of ANM values with both educational attainment and fluid intelligence between 41 and 49 years (Figure 4B), implying a genetic relationship between later reproduction, higher education, and later ANM for women before the age of 50. Interestingly, these genetic correlations also significantly change in sign for women whose ANM occurred after age 53, with a significant negative genetic correlation of ANM with educational attainment and fluid intelligence (Figure 4B), implying delayed reproduction and high educational attainment are associated with reproductive senescence post-age 50.

In a further follow-up analysis, we used MR, which utilizes the randomized inheritance of genetic variations in the population, to estimate the potential causal effect a modifiable risk factor or exposure has on a health-related outcome of interest. We used menopause at different ages as an exposure in five different MR methods (weighted median, inverse-variance weighted, simple mode, weighted mode, and MR-Egger; see Table S10) found in the “TwoSampleMR” R package. Note here again that effect-size estimates for ANM are calculated on the menopause hazard scale, and thus, to ease interpretation, we flip the sign of the estimated potential causal effect to give values on the observed ANM scale. When repeating the analysis for each varying age of our exposure, we find changes in the magnitude of the potential causal effect with age for educational attainment, leiomyoma, oestradiol, and neutrophil count (Figure 4C).

## Discussion

Taken together, we find that the majority of ANM genetic associations display some form of age specificity in their effects. In turn, that translates into the associations being differentially enriched in different biological pathways across ages, which then leads to different genetic associations of ANM and other health indicators and outcomes depending upon the timing of ANM, with different potential statistical causal relationships.

We find evidence that prolonged and delayed reproduction are genetically associated with reproductive senescence post-age 50 as the genetic correlations significantly change in the sign for women whose ANM occurred after age 53, with a significant negative genetic correlation of ANM with age at last birth and age at first birth (Figure 4B). Similarly, we find genetic correlations between ANM and educational attainment or fluid intelligence significantly turn negative for women after age 53. The latter patterns of changing genetic correlation may simply reflect changes in schooling opportunities or system. Furthermore, both age at first and last birth show a similar, but stronger, pattern, and it may be the underlying factor acting as a confounder in the education-ANM relationship.

Complementing the results from genetic correlations, our enrichment analysis results (Figure 3) show early ANM genetic associations being enriched for genes differentially expressed in female reproductive organs, oocytes, and DNA damage-repair mechanisms. Hence, our findings suggest that at the genetic level, breast cancer risk, hormone levels, and ANM are linked prior to age 50. The patterns observed in the MR analyses largely reflect those of the genetic correlations described above, but here we find little evidence for a causal relationship between ANM and breast cancer, heart failure, age at first or last birth, or hypothyroidism (Figure 4C). That implies that genetic correlation estimates likely reflect reverse causation or the presence of heritable confounders of the trait pairs.

Our enrichment analysis findings support a link between DNA damage-repair genes and repair and surveillance for the development of oocytes for early-ANM women. The size of the initial oocyte pool at birth, along with the rate of atresia, influences the age at which the oocyte pool is depleted. The meiosis that occurs in oocytes necessitates programmed double-stranded breaks (DSBs) that must be repaired through the homologous recombination pathway, with oocytes that do not properly repair DSBs after this first phase of meiosis undergoing apoptosis. Here, early-ANM-associated common variants are enriched at loci, harboring genes involved in the DNA repair and replication checkpoint processes, such as RNA polymerase II, histone methyltransferase complex, and histone acetylation (Figure 3). One-carbon metabolism has the ability to regulate the estrus cycle and modulate the initiation of reproductive senescence through the loss of methyl-donor production needed to properly maintain the epigenome. Our results support the existence of this mechanism as early-menopause associations are enriched in pathways associated with the hypothalamic-pituitary-gonadal (HPG) axis and with methylation in the nucleosome, with later-menopausal genetic associations showing no evidence of enrichment in these pathways (Figure 3). In humans, it has been suggested that postmenopausal women exhibit accelerated aging compared with premenopausal women of the same biological age.^27^ However, the cause-effect relationship between epigenetic changes and reproductive senescence remains unclear, and our results imply early-ANM women may have a methylation pattern associated with one-carbon metabolism that differs from the general population. Generally, our follow-up analyses support previous studies,^9^^,^^28^ but we demonstrate that almost all underlying pathways associated with variation in ANM act in an age-specific manner.

There are several important caveats to our study. First, we have assumed that the effect size can only change linearly with age, whereas in reality, they could consist of more complicated patterns that could be captured with piece-wise exponential models. However, introducing many more parameters on a genome-wide scale would lead to a high multiple-testing burden, potentially hampering the capability to detect the actual signal. Furthermore, especially for traits with a moderate range of values (90% of the observed ANM happens between ages 45 to 55, Figure S1), introducing many parameters could lead to overfitting. Therefore, we find that, especially in the context of traits such as ANM, assuming a linear effect change is a suitable compromise between the added value of learning new information about effect change and limiting the model complexity without damaging the statistical power. Nevertheless, the analyses presented here represent a first step, and we encourage specifying different functional forms for the effect size, preferably for traits with a broader range of values or a reasonable prior guess. Similarly, we have refrained from drawing conclusions about the causal effect change tendencies (e.g., linear vs. non-linear) as analyses claiming statistically significant non-linearity would be under-powered.

Second, the current implementation of the model is not computationally efficient, and to handle the computational burden we have utilized the computational resources of two universities to produce these results. Our objective was simply to highlight the existence of changing genetic relationships between phenotypes and health outcomes across the lifespan. Although it is possible to make marginal analyses embarrassingly parallel, it is inherently time consuming to fit a CAMP model. Scaling the inference requires new research into novel algorithms for computationally heavy high-dimensional statistical problems of this kind.

Finally, our study focused only on European ancestry individuals in the UK and Estonian Biobanks, and future analyses must take into account populations with more diverse ancestries to get a fuller picture of the genetic architecture of ANM across the globe. This requires research into statistical models that are capable of learning both shared and unique age-dependent effect sizes across populations, and it requires large-scale data to be collected from worldwide populations.

In summary, we propose an analysis approach for GWASs of age-at-onset phenotypes using a two-stage mixed linear-association model, where marker effect sizes are estimated using a CAMP model. Our approach provides a better understanding of the genetic basis of ANM and applies to any form of time-to-event phenotype.

## Data Availability

Age-specific summary statistic estimates are released publicly on Dryad: https://doi.org/10.5061/dryad.nvx0k6dx5. The BayesW model was executed with the software Hydra, with full open source code available at https://github.com/medical-genomics-group/hydra.^29^ The scripts used to execute CAMP model are available at https://github.com/svenojavee/CAMP. R version 4.2.1 is available at https://www.r-project.org/.

## Supplemental information

Document S1. Figures S1–S6 and Table S1

Table S2. Pathway enrichment analysis for age 41

Table S3. Pathway enrichment analysis for age 43

Table S4. Pathway enrichment analysis for age 45

Table S5. Pathway enrichment analysis for age 47

Table S6. Pathway enrichment analysis for age 49

Table S7. Pathway enrichment analysis for age 51

Table S8. Pathway enrichment analysis for age 53

Table S9. Pathway enrichment analysis for age 55

Table S10. Mendelian randomization analysis results

Table S11. Summary statistics used in Mendelian randomization analysis

Document S2. Article plus supplemental information

## References

[1] Ojavee S.E., Kousathanas A., Trejo Banos D., Orliac E.J., Patxot M., Läll K., Mägi R., Fischer K., Kutalik Z., Robinson M.R. (2021). Genomic architecture and prediction of censored time-to-event phenotypes with a bayesian genome-wide analysis. Nat. Commun..

[2] Pedersen E.M., Agerbo E., Plana-Ripoll O., Grove J., Dreier J.W., Musliner K.L., Bækvad-Hansen M., Athanasiadis G., Schork A., Bybjerg-Grauholm J. (2022). Accounting for age of onset and family history improves power in genome-wide association studies. Am. J. Hum. Genet..

[3] Shin J., Han K., Jung J.-H., Park H.J., Kim W., Huh Y., Kim Y.-H., Kim D.-H., Kim S.M., Choi Y.S. (2022). Age at menopause and risk of heart failure and atrial fibrillation: a nationwide cohort study. Eur. Heart J..

[4] Nash Z., Al-Wattar B.H., Davies M. (2022).

[5] Collaborative Group on Hormonal Factors in Breast Cancer (2012). Collaborative Group on Hormonal Factors in Breast Cancer and others. Menarche, menopause, and breast cancer risk: individual participant meta-analysis, including 118 964 women with breast cancer from 117 epidemiological studies. Lancet Oncol..

[6] Murabito J.M., Yang Q., Fox C., Wilson P.W.F., Cupples L.A. (2005). Heritability of age at natural menopause in the framingham heart study. J. Clin. Endocrinol. Metab..

[7] Stolk L., Perry J.R.B., Chasman D.I., He C., Mangino M., Sulem P., Barbalic M., Broer L., Byrne E.M., Ernst F. (2012). Meta-analyses identify 13 loci associated with age at menopause and highlight dna repair and immune pathways. Nat. Genet..

[8] Day F.R., Ruth K.S., Thompson D.J., Lunetta K.L., Pervjakova N., Chasman D.I., Stolk L., Finucane H.K., Sulem P., Bulik-Sullivan B. (2015). Large-scale genomic analyses link reproductive aging to hypothalamic signaling, breast cancer susceptibility and brca1-mediated dna repair. Nat. Genet..

[9] Ruth K.S., Day F.R., Hussain J., Martínez-Marchal A., Aiken C.E., Azad A., Thompson D.J., Knoblochova L., Abe H., Tarry-Adkins J.L. (2021). Genetic insights into biological mechanisms governing human ovarian ageing. Nature.

[10] Robinson M.R., English G., Moser G., Lloyd-Jones L.R., Triplett M.A., Zhu Z., Nolte I.M., van Vliet-Ostaptchouk J.V., Snieder H., LifeLines Cohort Study (2017). Genotype–covariate interaction effects and the heritability of adult body mass index. Nat. Genet..

[11] Joshi P.K., Fischer K., Schraut K.E., Campbell H., Esko T., Wilson J.F. (2016). Variants near chrna3/5 and apoe have age-and sex-related effects on human lifespan. Nat. Commun..

[12] Patxot M., Banos D.T., Kousathanas A., Orliac E.J., Ojavee S.E., Moser G., Holloway A., Sidorenko J., Kutalik Z., Mägi R. (2021). Probabilistic inference of the genetic architecture underlying functional enrichment of complex traits. Nat. Commun..

[13] Tasa T., Krebs K., Kals M., Mägi R., Lauschke V.M., Haller T., Puurand T., Remm M., Esko T., Metspalu A. (2019). Genetic variation in the estonian population: pharmacogenomics study of adverse drug effects using electronic health records. Eur. J. Hum. Genet..

[14] Purcell S., Neale B., Todd-Brown K., Thomas L., Ferreira M.A.R., Bender D., Maller J., Sklar P., De Bakker P.I.W., Daly M.J., Sham P.C. (2007). Plink: a tool set for whole-genome association and population-based linkage analyses. Am. J. Hum. Genet..

[15] Yang J., Ferreira T., Morris A.P., Medland S.E., Madden P.A.F., Heath A.C., Martin N.G., Montgomery G.W., Genetic Investigation of ANthropometric Traits GIANT Consortium, DIAbetes Genetics Replication And Meta-analysis DIAGRAM Consortium, et al (2012). Conditional and joint multiple-snp analysis of gwas summary statistics identifies additional variants influencing complex traits. Nat. Genet..

[16] Yang J., Lee S.H., Goddard M.E., Visscher P.M. (2011). GCTA: a tool for genome-wide complex trait analysis. Am. J. Hum. Genet..

[17] Staley J.R., Blackshaw J., Kamat M.A., Ellis S., Surendran P., Sun B.B., Paul D.S., Freitag D., Burgess S., Danesh J. (2016). Butterworth. PhenoScanner: a database of human genotype–phenotype associations. Bioinformatics.

[18] Kamat M.A., Blackshaw J.A., Young R., Surendran P., Burgess S., Danesh J., Butterworth A.S., Staley J.R. (2019). PhenoScanner v2: an expanded tool for searching human genotype–phenotype associations. Bioinformatics.

[19] Bakker O.B., Claringbould A., Westra H.-J., Wiersma H., Boulogne F., Võsa U., Symmons S.M., Jonkers I.H., Franke L., Deelen P. (2021). Linking common and rare disease genetics through gene regulatory networks. medRxiv.

[20] Sanderson E., Glymour M.M., Holmes M.V., Kang H., Munafò M.R., Munafò M.R., Palmer T., Schooling C.M., Wallace C., Zhao Q., Smith G.D. (2022). Mendelian randomization. Nat. Rev. Methods Primers.

[21] Davey Smith G., Hemani G. (2014). Mendelian randomization: genetic anchors for causal inference in epidemiological studies. Hum. Mol. Genet..

[22] Hemani G. (2020). ieugwasr: R interface to the ieu gwas database api. R package version 0.1, 5.

[23] Hemani G., Zheng J., Elsworth B., Wade K.H., Haberland V., Baird D., Laurin C., Burgess S., Bowden J., Langdon R., Base Collaboration (2018). The mr-base platform supports systematic causal inference across the human phenome. Elife.

[24] Han B., Eskin E. (2011). Random-effects model aimed at discovering associations in meta-analysis of genome-wide association studies. Am. J. Hum. Genet..

[25] Jiang X., Holmes C., McVean G. (2021). The impact of age on genetic risk for common diseases. PLoS Genet..

[26] Winkler T.W., Justice A.E., Graff M., Barata L., Feitosa M.F., Chu S., Czajkowski J., Esko T., Fall T., Kilpeläinen T.O. (2015). The influence of age and sex on genetic associations with adult body size and shape: a large-scale genome-wide interaction study. PLoS Genet..

[27] Levine M.E., Lu A.T., Chen B.H., Hernandez D.G., Singleton A.B., Ferrucci L., Bandinelli S., Salfati E., Manson J.E., Quach A. (2016). Menopause accelerates biological aging. Proc. Natl. Acad. Sci. USA.

[28] Ward L.D., Parker M.M., Deaton A.M., Tu H.-C., Flynn-Carroll A.O., Hinkle G., Nioi P. (2022). Rare coding variants in dna damage repair genes associated with timing of natural menopause. HGG Adv..

[29] Robinson M. (2021). hydra (version v1.0). Zenodo.

