# Supplementary material for: Genetic insights into the age-specific biological mechanisms governing human ovarian aging
Source: Am J Hum Genet. 2023 Aug 4;110(9):1549–63. doi: 10.1016/j.ajhg.2023.07.006 (PMC10502738; doi:10.1016/j.ajhg.2023.07.006)
Supplement: Document S1. Figures S1–S6 and Table S1 [file mmc1.pdf]

The American Journal of Human Genetics, Volume *110*

Supplemental information

Genetic insights into the age-specific biological mechanisms governing human ovarian aging

Sven E. Ojavee, Liza Darrous, Marion Patxot, Kristi Läll, Krista Fischer, Reedik Mägi, Zoltan Kutalik, and Matthew R. Robinson

Supplementary Figures

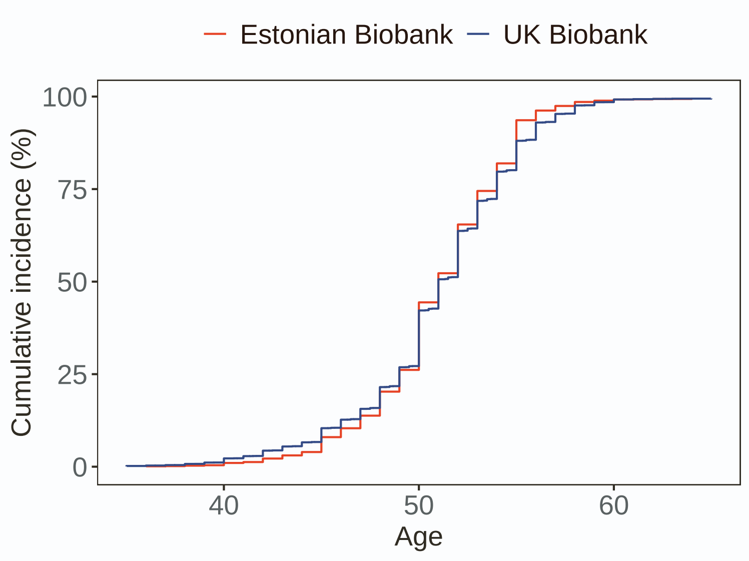

Figure S1. Cumulative incidence curves of menopause. Cumulative incidence curves take into account the competing risk of death. The distributions of age-at-menopause in the UK and Estonian Biobanks are relatively similar, although more menopauses happen somewhat earlier in UK Biobank than in the Estonian Biobank.

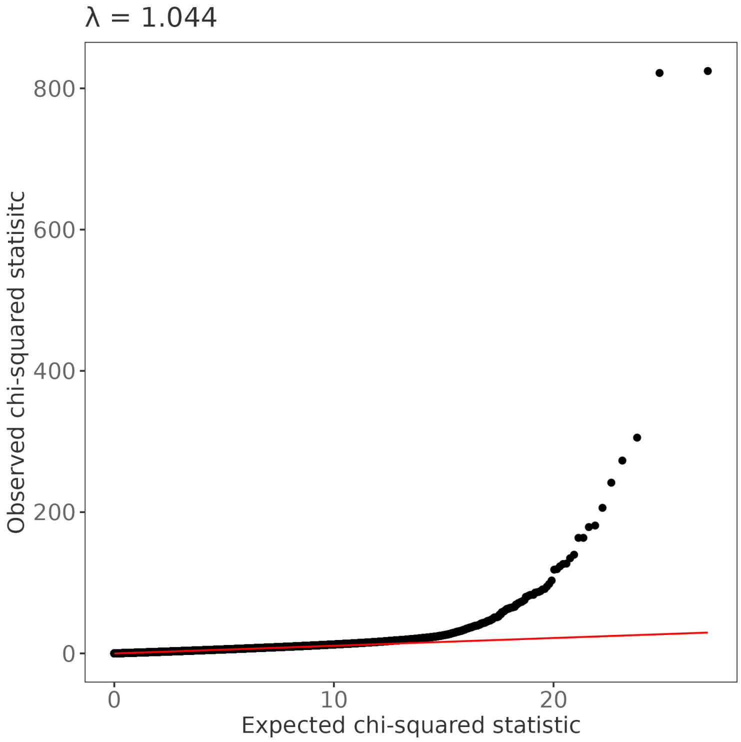

Figure S2. QQ plot of age-at-menopause GWAS using CAMP model. The x-axis uses the $\chi^2_{df=2}$ distribution quantiles as under the null hypothesis of no effect ($\beta^0 = 0$ and $\beta^1 = 0$) the test statistic follows that distribution. The genomic inflation factor is 1.044, median of $\chi^2_{df=2}$ distribution is 1.386.

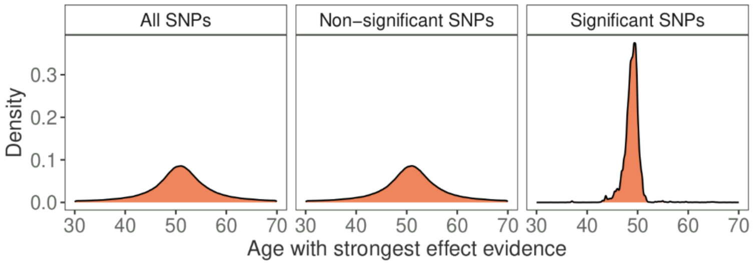

Figure S3. Distribution of ages with the most evidence for an effect size. Distribution of the ages when the maximum chi-squared statistic is achieved (calculated using Equation **??**) over all SNPs, over all non-significant SNPs and significant SNPs only. Even though the distributions have similar centres (median age of 51 and 49 for all SNPs and significant SNPs, respectively), the standard deviation of all SNP age distribution is 3.7 times higher than significant SNP age distribution.

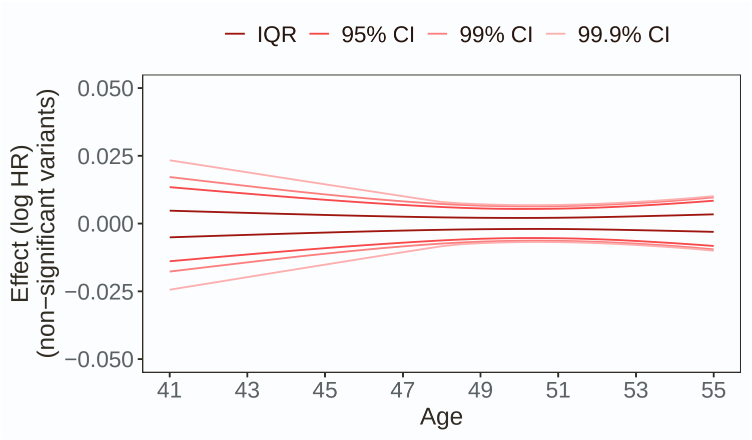

Figure S4. Effect sizes (log HR) for nominally non-significant effects by summarising the effects between the interquartile range, 95% CI, 99% CI or 99.9% CI. We observe that compared to the significant effect sizes, the non-significant effects are much smaller and do not vary much across ages. Nominally insignificant effects are defined as $p > 0.05$.

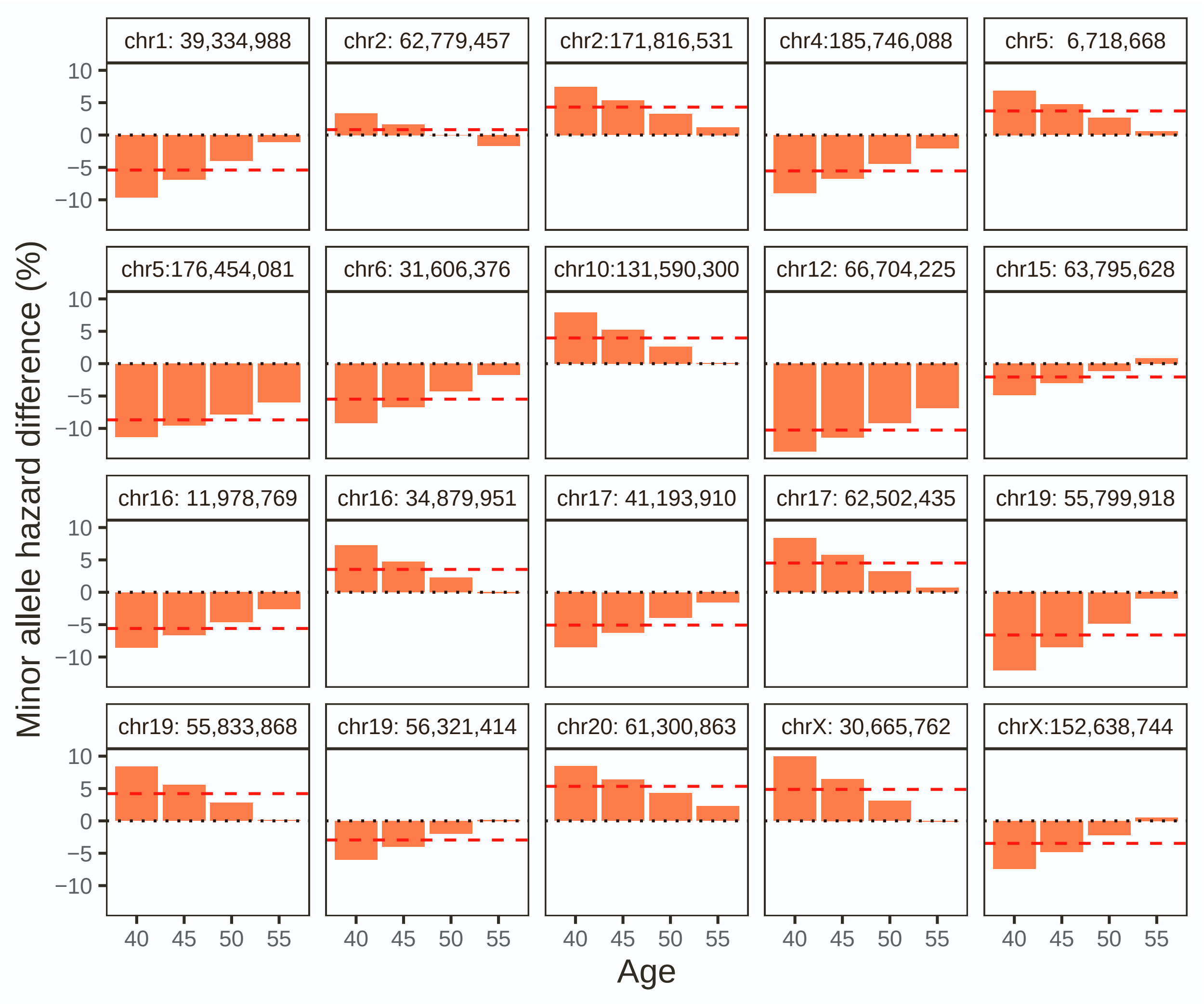

Figure S5. Change in hazard for the variants with a significant age-specific effects
Illustration of the effect size change for the variants with an age-specific effects in Table **??**.

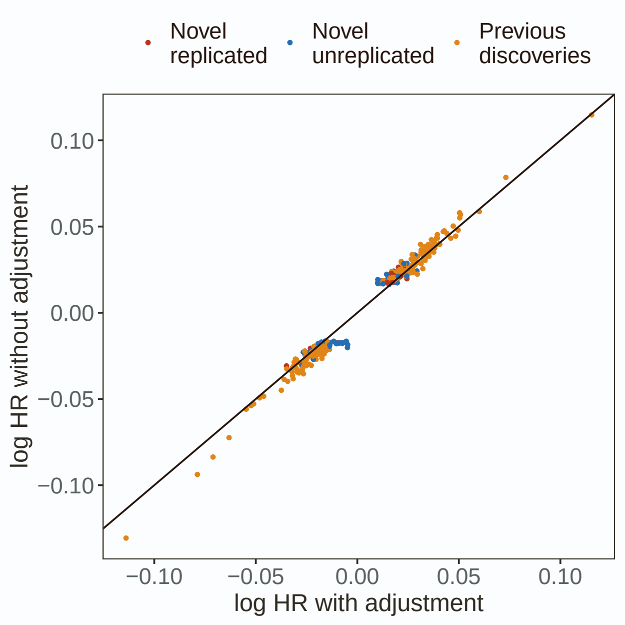

Figure S6. Sensitivity analysis for a model with and without adjusting hormone replacement therapy (HRT) as a time-varying covariate. Log hazard ratio effect size estimates for the 312 age-at-natural menopause (ANM) associations with (x-axis) and without (y-axis) HRT as covariate. The effect sizes shown are the age between 45-52 where the maximum χ^2 statistic value was obtained for either model. Novel replicated are the 19 SNPs that have not been menopause-associated before but are significant both in the CAMP and the Estonian Biobank analyses. Previous discoveries are the 226 SNPs that were menopause-associated before and are also significant in the CAMP analysis. Novel unreplicated are the 67 SNPs that are not been menopause-associated before but they are significant in the CAMP analysis without replication in the Estonian Biobank.

Supplementary Tables

SNP	Chr	Position	Eff/Oth	MAF	Nearest gene	Yearly effect change	Slope p-value	Strongest evidence p-value
rs115441717	1	17,587,645	A/G	0.005	PADI3	-0.0036	5.37×10^{-10}	7.51×10^{-10}
rs186475193	1	66,520,884	A/T	0.003	PDE4B	-0.0036	8.05×10^{-11}	1.30×10^{-10}
rs61813814	1	178,259,880	A/G	0.004	RASAL2	-0.0035	3.34×10^{-9}	3.18×10^{-7}
rs9662346	1	246,893,259	G/A	0.271	SCCPDH	0.0039	5.59×10^{-9}	1.20×10^{-3}
rs1406295	2	27,689,700	G/A	0.393	IFT172	-0.0038	5.21×10^{-9}	1.92×10^{-50}
rs2293269	2	48,844,968	C/G	0.352	GTF2A1L*	-0.0044	3.30×10^{-11}	1.79×10^{-20}
rs117292624	3	5,813,506	A/T	0.006	AC027119.1*	-0.0034	6.36×10^{-9}	3.66×10^{-8}
rs1317571	3	49,910,657	T/G	0.177	ACTBP13*	-0.0042	2.99×10^{-10}	5.27×10^{-12}
rs9818740	3	135,939,586	A/G	0.274	RP11-463H24.1*	-0.0042	1.35×10^{-10}	1.01×10^{-14}
rs149660889	4	7,769,181	C/T	0.006	AFAP1*	-0.0035	1.61×10^{-9}	3.36×10^{-7}
rs7661090	4	13,571,901	T/C	0.105	BOD1L1	-0.0040	3.45×10^{-10}	1.42×10^{-18}
rs11099599	4	84,367,050	A/G	0.492	HELQ	0.0048	3.28×10^{-13}	6.18×10^{-130}
rs116822956	5	6,790,631	G/T	0.024	RP11-332J15.1*	-0.0042	9.96×10^{-12}	3.42×10^{-3}
rs11737992	5	175,953,121	T/C	0.309	RNF44*	-0.0042	1.88×10^{-10}	1.58×10^{-30}
rs76706863	6	28,291,052	G/T	0.018	ZSCAN31*	-0.0036	3.73×10^{-9}	1.27×10^{-6}
rs138131857	6	29,654,098	A/G	0.018	ZFP57*	-0.0041	2.80×10^{-11}	1.66×10^{-10}
rs11752373	6	90,583,601	A/T	0.045	CASP8AP2	-0.0037	3.79×10^{-9}	3.22×10^{-6}
rs140020429	6	109,831,159	T/C	0.006	AK9	-0.0034	4.88×10^{-9}	2.77×10^{-6}
rs117109951	7	123,057,448	C/T	0.003	IQUB*	-0.0034	6.25×10^{-9}	1.06×10^{-6}
rs10093345	8	37,872,776	C/T	0.259	EIF4EBP1*	-0.0060	2.33×10^{-18}	3.02×10^{-98}
rs76637775	8	69,027,721	G/A	0.008	PREX2	-0.0036	4.68×10^{-9}	7.68×10^{-7}
rs138790299	8	80,383,022	G/A	0.003	RP11-758H6.1*	-0.0035	6.67×10^{-10}	3.18×10^{-7}
rs112136293	8	135,941,782	A/T	0.003	RP11-1057B8.2*	-0.0035	1.11×10^{-9}	1.24×10^{-6}
rs117273292	9	91,945,999	C/T	0.03	SECISBP2	-0.0037	1.72×10^{-9}	9.40×10^{-7}
rs113357099	10	75,065,684	A/G	0.083	TTC18	0.0041	3.57×10^{-9}	8.83×10^{-4}
rs79293542	10	97,809,854	C/T	0.034	CCNJ*	-0.0042	1.02×10^{-11}	1.80×10^{-26}
rs11245450	10	126,658,075	A/G	0.420	ZRANB1	-0.0039	3.67×10^{-9}	9.68×10^{-32}
rs655139	11	102,269,679	G/T	0.082	TMEM123	-0.0038	4.45×10^{-9}	2.49×10^{-2}
rs3782232	12	57,116,249	A/G	0.071	NACA	-0.0041	1.64×10^{-10}	5.04×10^{-64}
rs80256675	12	68,088,664	C/A	0.007	RP11-43N5.1*	-0.0036	2.10×10^{-9}	2.86×10^{-6}
rs11180609	12	76,040,392	C/T	0.063	RP11-114H23.1	-0.0040	4.60×10^{-10}	7.93×10^{-12}
rs145313906	12	83,821,558	A/T	0.003	RP11-384P14.1*	-0.0038	7.07×10^{-12}	5.35×10^{-12}
rs2954111	12	122,134,415	C/T	0.36	TMEM120B*	-0.0048	1.45×10^{-13}	2.45×10^{-17}
rs7322160	13	61,061,456	C/T	0.328	TDRD3	0.0045	1.81×10^{-11}	1.89×10^{-42}
rs146576015	16	60,155,191	G/A	0.010	RP11-430C1.1*	-0.0035	6.00×10^{-9}	4.52×10^{-8}
rs140438673	16	85,217,665	A/G	0.007	CTC-786C10.1	-0.0035	6.05×10^{-9}	2.29×10^{-8}
rs13331995	16	89,912,063	C/T	0.346	SPIRE2	-0.0045	7.59×10^{-12}	2.90×10^{-29}
rs62097189	18	32,191,404	G/A	0.003	DTNA	-0.0034	2.22×10^{-9}	1.13×10^{-12}
rs148228698	19	2,417,585	A/G	0.008	TMPRSS9	-0.0035	6.61×10^{-9}	2.50×10^{-7}
rs7253685	19	55,845,386	G/A	0.378	TMEM150B	-0.0067	2.25×10^{-24}	1.57×10^{-70}
rs16991615	20	5,948,227	A/G	0.066	MCM8	0.0091	1.13×10^{-36}	1.13×10^{-36}
rs5951636	X	21,841,633	C/T	0.377	MBTPS2*	-0.0041	3.11×10^{-10}	3.69×10^{-18}
rs12559662	X	44,155,570	T/C	0.101	EFHC2	-0.0038	3.69×10^{-9}	1.06×10^{-14}

Table S1. Regions with a genome-wide significant age-specific effect on age-at-menopause not replicated in the Estonian Biobank. Although these SNPs did not have a significant slope in the replication data set, the genome-wide significant non-zero slopes indicate that the constant effect assumption might be too stringent when analysing the effect of these variants. For each SNP we tested the significance of the slope parameter using the CAMP model. The results were then LD clumped such that the index SNPs would have a p-value below $5 \cdot 10^{-8}$ and SNPs could be added to a clump if they were 1Mb from the index SNP, they were correlated with $r^2 > 0.05$ and they were nominally significant ($p < 0.05$). We then used the COJO method from the GCTA software (see Methods) to find clumps with independent signals by conducting stepwise selection of index SNPs in 1Mb window and we considered SNPs independent if they had a p-value below $5 \cdot 10^{-8}$ in the joint model. The candidates for significant slope were replicated in the Estonian Biobank. Replication was defined as p-value being lower than 0.05 and the direction of the effect size same in both the original analysis and the replication analysis. The effect size estimates are reported on the log hazard scale. The column Nearest gene is mapped from the SNP using ANNOVAR software, * in that column denotes intergenic regions; chromosome X nearest gene was determined by using the UCSC Genome Browser. The column Strongest evidence p-value indicates the p-value at age when there is strongest evidence for an effect.
